# Phytotoxic effects of *Acacia saligna* dry leachates on germination, seedling growth, photosynthetic performance, and gene expression of economically important crops

**DOI:** 10.7717/peerj.13623

**Published:** 2022-08-02

**Authors:** Haifa Abdulaziz Sakit ALHaithloul, Muhammad Ishfaq Khan, Arafa Musa, Mohammed M. Ghoneim, Ayshah Aysh ALrashidi, Imtiaz Khan, Ehab Azab, Adil A. Gobouri, Mahmoud R. Sofy, Mohamed El-Sherbiny, Mona H. Soliman

**Affiliations:** 1 Biology Department, College of Science, Jouf University, Sakaka, Saudi Arabia; 2 Department of Weed Science and Botany, University of Agriculture Peshawar, Peshawar, Pakistan; 3 Department of Pharmacognosy, College of Pharmacy, Jouf University, Sakaka, Aljouf, Saudi Arabia; 4 Pharmacognosy and Medicinal Plants Department, Faculty of Pharmacy (Boys), Al-Azhar University, Cairo, Egypt; 5 Department of Pharmacy Practice, College of Pharmacy, Al Maarefa University, Ad Diriyah, Saudi Arabia; 6 Department of Biology, Faculty of Science, University of Hail, Hail, Saudi Arabia; 7 Department of Food Science and Nutrition, College of Sciences, Taif University, Taif, Saudi Arabia; 8 Department of Chemistry, College of Science, Taif University, Taif, Saudi Arabia; 9 Botany and Microbiology Department, Faculty of Science, Al-Azhar University, Cairo, Egypt; 10 Department of Basic Medical Sciences, College of Medicine, AlMaarefa University, Riyadh, Saudi Arabia; 11 Department of Anatomy, Faculty of Medicine, Mansoura University, Mansoura, Egypt; 12 Botany and Microbiology Department, Faculty of Science, Cairo University, Giza, Egypt; 13 Biology Department, Faculty of Science, Taibah University, Yanbu, Medina, Saudi Arabia

**Keywords:** *Acacia saligna*, Allelopathy, Gene expression, Osmolytes, Secondary metabolism, Allelopathy/phytotoxicity

## Abstract

The influence of dry leachates of *Acasia saligna* was tested on the seedling growth, photosynthesis, biochemical attributes, and gene expression of the economically important crops, including wheat (*Triticum aestivum* L.), radish (*Raphanus sativus* L.), barley (*Hordeum vulgare* L.) and arugula (*Eruca sativa* L.). Different concentrations (5%, 10%, 15%, 20%, and 25%) of stem extract (SE) and leaf extract (LE) of *A. saligna* were prepared, and seedlings were allowed to grow in Petri plates for 8 days. The results showed that all plant species exhibited reduced germination rate, plant height, and fresh and dry weight due to leachates extracts of *A. saligna*. Moreover, the activities of antioxidant enzymes, including superoxide dismutase (SOD), catalase (CAT), and ascorbate peroxidase (APX), exhibited differential regulation due to the extract treatment. The SOD was increased with increasing the concentration of extracts, while CAT and APX activities were decreased with increasing the extract concentrations. In addition, leachate extract treatment decrease chlorophyll content, photosynthesis, PSII activity, and water use efficiency, with evident effects at their higher concentrations. Furthermore, the content of proline, sugars, protein, total phenols, and flavonoids were reduced considerably due to leachates extract treatments. Furthermore, seedlings treated with high concentrations of LE increased the expression of genes. The present results lead to the conclusion that *A. saligna* contains significant allelochemicals that interfere with the growth and development of the tested crop species and reduced the crops biomass and negatively affected other related parameters. However, further studies are suggested to determine the isolation and purification of the active compounds present in *A. saligna* extracts.

## Introduction

Numerous primary and secondary compounds are produced by plants that are consequently released into their environment either through volatilization, root exudates, or decomposition and leaching by the leftover residues. The released compounds or metabolites can impart damaging or beneficial effects on the plants growing in the vicinity, referred to as the allelopathic effect (Grutters et al., 2017; Hadacek, 2002). The damaging influence of these released secondary compounds on the other plant species is considered as allelochemical stress (Abu-Shahba et al., 2022) and categorized as biotic stress. On its positive aspects, allelopathy leads to an organic approach through its natural means (Seufert et al., 2019). On the other hand, the effects of allelochemicals are diverse and intense, consequently altering the vegetation pattern, crop and weed growth, and productivity (Cheng & Cheng, 2015). Furthermore, it has been reported that plant allelochemicals affect germination, biochemical and physiological attributes and influence other plants’ mineral uptake and assimilation. Resulting in reduced yield, and this trait can sometimes be used against the associated weeds of the crops as a natural herbicide (Tomar & Agarwal, 2013).

Allelochemicals/phytochemicals mediated a drastic decrease in growth associated with the upsurge in reactive oxygen species (ROS), which include hydroxyl, hydrogen peroxide, and superoxide formed at different sites within different organelles (Lara-NuÑez et al., 2006). Increased ROS cause calcium-dependent signaling cascade initiating changes at genetic and molecular levels (Bais et al., 2003). Excess ROS accumulation leads to oxidative harm to membranes, nucleic acids, and proteins (Ahanger et al., 2017). Such oxidative effects significantly damage root growth and proliferation, reducing water uptake, photosynthesis, and assimilate production, reducing crop productivity (Lara-NuÑez et al., 2006). Plant species are armed with defense mechanisms that coordinately assist plants in countering any damaging effect. The antioxidant system, osmolyte accumulation, and gene expression have unique roles in eliminating toxic radicals (Ahanger et al., 2017). Antioxidant structures include enzymatic and non-enzymatic constituents that maintain cells’ structural and functional integrity by scavenging ROS. Osmolytes prevent turgor loss, scavenge ROS and protect the enzyme activity (Ahanger et al., 2014; Sivakumar et al., 2002). The changes drive loss of photosynthetic activity and growth reduction at the molecular levels (Abdelmigid & Morsi, 2017).

*Acacia saligna* that belongs to the family Fabaceae is a small tree and has a worldwide distribution, commonly known as orange wattle, blue-leafed wattle, golden wreath wattle, coojong, *etc*. It is a small tree with dense spreading short stem and a howling habit. It has a phyllode with a nectar gland secreting sugary fluid that usually attracts ants. As a natural colonizer or invasive potential, *A. saligna* can grow in disturbed soils like new roads. It has been reported to contain a greater concentration of phenols, flavonoids, and terpenes (El-Toumy et al., 2010; Lin & Chang, 2013). Therefore, its continuous planting along the roadsides and agricultural lands can lead to the excess accumulation of phytochemicals present in it, posing a threat to the crop plants’ productivity around this plant.

*Triticum aestivum* and *Hordeum Vulgar* are important staple food crops consumed throughout the world belonging to the family Poaceae, while *Raphanus sativus *and, *Eruca Sativa* are important vegetable crops belonging to the Brassicaceae family. All these plant species are rich in proteins and minerals and form an important source of critical metabolites. Excessive planting of *A. saligna* leads to accumulation of allelochemicals in agricultural soils that can drastically influence their growth as well as productivity, thus affecting the lifestyle of the world populace. The present study was envisioned that allelochemical extracts of *A. saligna* can reduce the growth of the above-mentioned plants by altering photosynthesis, biochemical attributes, expression of genes. To explore the phenomenon of allelopathy/ phytotoxicity and the potency of the *A. saligna* extracts for future studies to closely know about the effect of natural compounds that could be further explored.

In the present study stem extracts and root extracts of *Acacia saligna* were tested to know their effects on the important physiological attributes, *e.g*., ROS, CAT, APX, water use efficiency, photosynthesis, and PS-II activity on economically important crops. Moreover, biochemical parameters and secondary metabolites such as proline, sugars, total phenols, and total flavonoids and gene expressions were also studied in instant trials, which are novel approaches regarding *Acacia sligna* extracts. Leaves and stem of *A. saligna* were collected from the El Jouf area Kingdom of Saudi Arabia.

## Materials and Methods

### Plant extract preparation

Leaves and stem tissues of *A. saligna* tree were harvested and washed with distilled water and left to dry at room temperature in a shaded place for several days till complete dryness. The dried samples were ground well to pass a 1 mM screen, and then stored at 4 °C. Different concentrations *i.e*., 5, 10, 15, 20, 25 gm of dry powdered *A. saligna* leaves and stem were added to 100 mL distilled water and subjected to rotary evaporator for 48 h at 40 °C. Thereafter extracts were filtered through muslin cloth followed by Whatman No. 1 filter article and final volume was made upto 100 mL. Extract was stored at 4 °C until further use (Tomar, Sharma & Agarwal, 2015).

### Crop growth and experimental treatments

Seeds of wheat (*Triticum aestivum*), barley (*Hordeum vulgare*), radish (*Raphanus sativus*), and arugula *(Eruca Sativa*) were treated with NaOCl (5%). Sterilized seeds were placed in the Petri dishes with 9 cm size on two layers of filter paper Whatman No. 1 and wetted with 5 mL of extract of each *A. saligna* leaf or stem concentration. Control was supplied with 5 mL of distilled water. In addition, 3 mL of 10% Hoagland solution was added to each treatment every 2 days to overcome the nutrient deficiency. Petri plates were arranged in a completely randomized design with four replications and kept at room temperature (25 °C) for 8 days, with 70% humidity with 12 h photoperiod. The seeds were divided into two groups, and each group was divided into five subgroups as follows:

T1: control (5 mL of distilled water)

T2: 5% SE as 5 g/L stem extract (SE) of *A. Saligna*

T3: 10% SE as 10 g/L stem extract (SE) of *A. Saligna*

T4: 15% SE as 15 g/L stem extract (SE) of *A. Saligna*

T5: 20% SE as 20 g/L stem extract (SE) of *A. Saligna*

T6: 25% SE as 25 g/L stem extract (SE) of *A. Saligna*

T7: 5% LE as 5 g/L leaf extract (LE) of *A. Saligna*

T8: 10% LE as 10 g/L leaf extract (LE) of *A. Saligna*

T9: 15% LE as 15 g/L leaf extract (LE) of *A. Saligna*

T10: 20% LE as 20 g/L leaf extract (LE) of *A. Saligna*

T11: 25% LE as 25 g/L leaf extract (LE) of *A. Saligna*

The growth and other different parameters were analyzed after eight days, and the details of experimentation done are given here as under:

#### Germination parameters

Seeds showing a radical of 0.5 cm was considered as germinated seed. The following formulas calculated calculation of germination index, 50%, and germination percentage:

${\rm Germination\; percentage\; }\left( {{\rm GP}} \right) = \displaystyle{{{\rm No}.{\rm \; of\; germinated\; seeds}} \over {{\rm total\; no}.{\rm \; of\; seeds\; planted}}}{\rm \; } \times {\rm \; }100$

(1) $$\rm{Germination\; index \;(GI) = (G1/1) + (G2/2) + .........+ (Gx / x) }$$

where G = germination day

X = days of germination

#### Morphological traits

Morphological traits, including plant height, fresh and dry biomass, were examined after 8 days of extract treatment. An electronic weight balance was used to measure fresh shoots and roots, followed by oven-dried plant tissues at 70 °C for 48 h to calculate the dry weight.

### Chlorophyll content and photosynthetic characteristics

In acetone leaf samples of 100 mg were extracted, and the supernatant’s absorbance was recorded using a spectrophotometer (Genesys 10S UV-VIS, Thermo Fisher Scientific, Waltham, MA, USA) at 622, 664, and 440 nm. To calculate leaf chlorophyll (Chl.) Content (Lichtenthaler, 1987). The net photosynthetic (Pn) value was measured using an infrared gas analyzer system (TPS-2, USA) in fully expanded leaves between 09:00 and 11:00 AM. Fv/Fm was measured using a chlorophyll fluorometer (PAM 2500, Germany). Used water efficiency can be obtained by net photosynthetic /transpiration (Pn/Tr) (Sofy et al., 2021a).

### Biochemical analysis

#### Lipid peroxidation, hydrogen peroxide, and electrolyte leakage

Malonaldehyde (MDA) was estimated by following the method of Heath & Packer (1968). A molar coefficient of 155 mmol^−1^ cm^−1^ was used for calculation and expressed as nmol g^−1^ FW.

For hydrogen peroxide, fresh 100 mg tissues were homogenized in 2 mL of 0.1% trichloroacetic acid (TCA) solution, and the homogenate was centrifuged at 12,000*g* for 15 min. Supernatant (0.5 mL) was mixed with 0.5 mL of potassium phosphate buffer (10 mM, pH 7.0) and 1 mL of potassium iodide (1 M). Absorbance was read at 390 nm using spectrophotometer (Shimadzu, Kyoto, Japan), and the amount of H_2_O_2_ was calculated from the standard curve (Dawood et al., 2022).

The method described by Dionisio-Sese & Tobita (1998) was used to examine electrolyte leakage. Twenty fresh leaf discs’ electrical conductivity (EC0) were briefly determined after floating them in the test tube with 10 mL distilled water. The samples were then boiled for 20 min and 10 min at 50 °C and 100 °C, respectively, and electrical conductivities (EC1 and EC2, respectively) were recorded. Digital conductivity meter was used for taking readings, finally, the formula given below was used to estimate the electrolyte leakage.

$EL\left( {\rm \% } \right) = \displaystyle{{\left( {EC1 - ECo} \right)} \over {\left( {EC2 - ECo} \right)}} \times 100$

#### Proline, total soluble sugars, total phenolic, flavonoids content, and protein

For proline estimation, the method of Bates, Waldren & Teare (1973) was used. Plants extracted in sulfosalicylic acid (3%) followed by centrifugation at 10,000 rpm for 10 min. The mixture containing 2 mL supernatant, 2 mL ninhydrin reagent, and 2 mL glacial acetic acid were heated at 100 °C for 1 h. After cooling, proline was extracted with toluene, and absorbance was read at 520 nm using spectrophotometer (Shimadzu, Kyoto, Japan).

The method of Irigoyen, Einerich & Sánchez-Díaz (1992) was used to quantify the soluble sugars. One hundred mg of finely powdered sample was extracted in ethanol (80%), and total soluble sugar content was examined using the anthrone reagent. The calculation was carried using calibration curves of glucose.

For estimation of total phenol content method of Singleton, Orthofer & Lamuela-Raventós (1999) was used. Tissue was extracted in ethanol, and the extract was reacted with folin-Ciocalteu reagent and Na_2_CO_3_. Gallic acid was used as a standard.

Flavonoids were extracted in methanol, and the homogenate was centrifuged at 10,000 rpm for 10 min. The supernatant was reacted with NaNO_2_ and AlCl_3,_ followed by the addition of NaOH after 5 min. Absorbance was recorded at 510 nm, and the calculations were done using the standard curve of catching (Zhishen, Mengcheng & Jianming, 1999).

A total of 100 mg fresh leaf tissue was homogenized in phosphate buffer (0.1 M, pH 7.0) using cooled mortar and pestle for protein estimation. The concentration of protein and absorbance was read at 595 nm, according to Bradford (1976). Bovine serum albumin was used as the standard.

#### Assay of antioxidant enzymes

For extraction of enzymes, 1 g fresh leaf tissue was grounded by using mortar and pestle in 5 mL ice-cold extraction buffer (50 mM potassium phosphate buffer, pH 7.0), containing 1 mM EDTA and 2% polyvinyl pyrrolidone (PVP). The homogenate was centrifuged at 18,000 rpm for 30 min at 4 °C, and the supernatant was collected and used for enzyme assay.

The method by Beyer & Fridovich (1987) was used to assay the superoxide dismutase (SOD, EC 1.15.1.1) activity. The assay mixture contained 100 mM phosphate buffer (pH 7.4), methionine, nitrobluetetrazolium (NBT), 2 µM riboflavin, 0.1 mM EDTA, and enzyme extract. The mixture was incubated under light for 15 min, and photo reduction of NBT was read at 560 nm against the dark incubated samples. For assaying catalase (CAT, EC 1.11.1.6) activity method of Dhindsa, Plumb-Dhindsa & Thorpe (1981) was adopted, and the decrease in optical density was measured at 240 nm for 2 min in a reaction mixture containing 50 mM phosphate buffer (pH 7.0), H_2_O_2_, and enzyme extract. For calculation extinction coefficient of 0.036 mM^−LCM−1^ was used. The activity of APX was determined in an assay mixture containing 0.1 mL enzyme, 1 mL of 100 mM potassium phosphate buffer (pH 7.0), 0.1 mM EDTA, ascorbate, and H_2_O_2_. The reduction in absorbance was recorded at 290 nm for 2 min (Nakano & Asada, 1981). Antioxidant enzymes activities were expressed as U mg^−1^ protein.

### Total mRNA extraction and RT-PCR studies

According to the manufacturer’s protocol, a total RNA extraction kit (Sigma-Aldrich, St. Louis, MO, USA) was used for total mRNA extraction that was isolated from 0.1 g of leaf tissue. The isolated RNA was spectrophotometrically quantified and examined on a 1% agarose gel. RNA reverse transcription was carried out by following our previous method (Alhaithloul, 2019). The reaction mixture were included 10 as oligodT primer (10 pml/µL), 2.5 µL 5X buffer, 2.5 µL MgCl_2_, 2.5 µL 2.5 mMdNTPs, 4 µl from oligo (dT), 0.2 µL (5 unit µL^−1^) reverse transcriptase (Promega, Madison, WI, USA) and 2.5 µL RNA. The RT-PCR amplification was carried out in a thermal cycler PCR set to 42 °C for 1 h and 72 °C for 20 min.

#### qReal-time PCR

Table 1 lists the primer sequences used in quantitative real-time PCR (qRT-PCR) for gene expression investigation of two genes, CYP (cytochrome P450) and glutathione S-transferase (GST). A total of 20 µL of reaction volume was used, which included 2 µL of template, 10 µL of SYBR Green Master Mix, 2 µL of reverse primer, 2 µL of forwarding primer, and deionized water. The following conditions were used for PCR assays: 95 °C for 15 min, followed by 40 cycles of 95 °C for 30 s and 60 °C for 30 s. CT values were calculated using the CT of each sample (target gene CT subtracted from actin gene CT). The 2^−ΔΔCt^ method was used to determine relative gene expression (Togawa et al., 2008).

**Table 1:** Primers used for quantitative real-time PCR for cytochrome and S-transferase genes.

Genes			Primer sequence (5′–3′)
GST	TtGSTU2	F	5-GTGTGCTGGCTCAGTTAG-3
	TtGSTU3	R	5-GCATCAAGCGAGCCGAAAC-3
CYP 450	*CYP72A*	F	5- CAGTGATGACTTGCTAGGATTG-3
		R	5-CATGCTGAGCAGAATTAGTGTC-3
	CYP81A	F	5-AGGGGAGACGGGATGCTGG-3
		R	5-TTGGGCATGGTGATCCCTGG-3
Ref.	β-Actin	F	5-GGTTCACTTGAAGGGTGGTG-3
		R	5-TGAGGTGTACCTGTCCTCGTT

### Statistical examination

The experimental design was completely randomized, and statistical analysis was conducted with the statistical software SPSS (Statistical Package for the Social Science Version 26.0) (Gomez & Gomez, 1984). A one-way ANOVA with *post hoc* test variance analysis from Fisher’s test with Levine’s sample parametric distribution was used for the quantitative analysis. The confidence interval was set to 95%, and the negotiated margin for error was fixed at 5%. All graphs were done with Graph Pad Prism 8.

## Results

### Germination percentage and mean germination index

**Figure 1:**
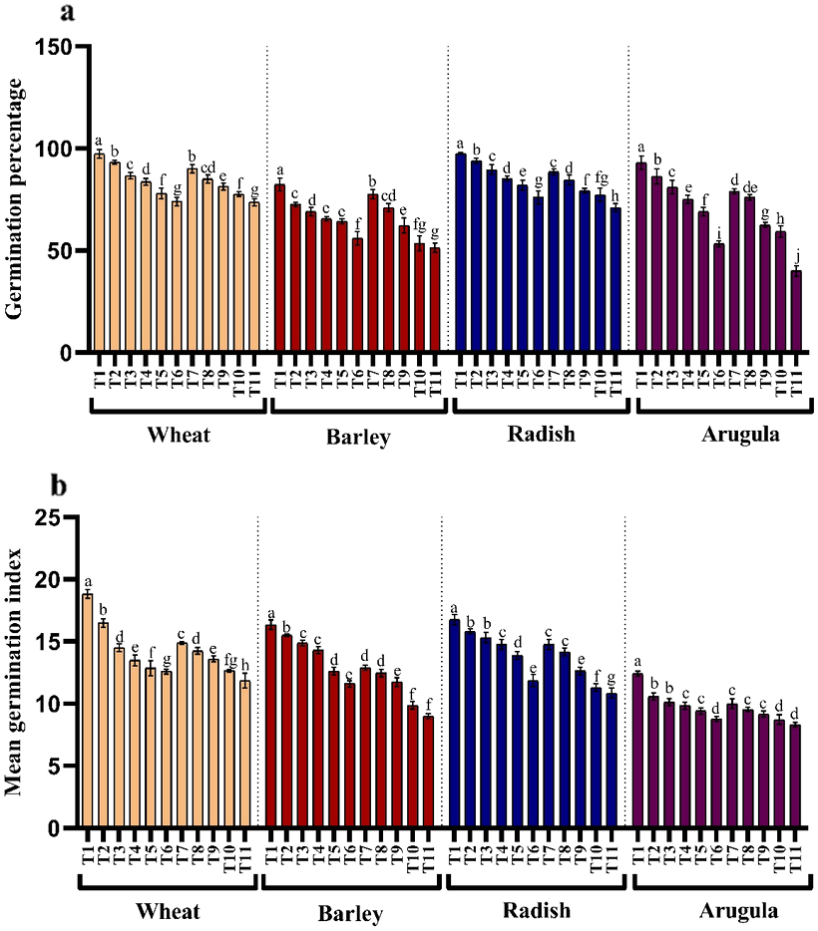
Effect of *A. Saligna* stem extracts (SE) and leaf extracts (LE). (A) germination percentage and (B) mean germination index in wheat, barley, radish, and arugula plant. Data is mean (±SE) of three replicates, and different lowercase letters denote significant difference at *P* < 0.05. T1: control, T2: 5% SE, T3: 10% SE, T4: 15% SE, T5: 20% SE, T6: 25% SE, T7: 5% LE, T8: 10% LE, T9: 15% LE, T10: 20% LE, T11: 25% LE.

The influence of stem and leaf extracts on the germination percentage and mean germination index is shown in Figs. 1A and 1B. The germination and mean germination index reduced with increasing extract concentration, attaining maximal decrease with higher concentration, *i.e*., 25%. Relative to control, percent decrease in germination percentage was 23.97, 31.96, 22.13, and 42.69% due to stem extract (25% SE) and 24.32%, 37.67%, 27.25%, and 56.99% due to leaf extract (25% LE) for *T. aestivum, H*. *vulgare*, *R. sativus*, and *E. Sativa* seedlings, respectively. However, lower concentration (5%) of both stem and leaf extracts of *A. saligna* did not impart such a significant reduction in germination attributes of all four plant species. Moreover, the treatment with four plant species with 25% SE or 25% LE led to a higher decrease in the germination index than the control plant (Fig. 1B).

### Morphological growth parameters

**Figure 2:**
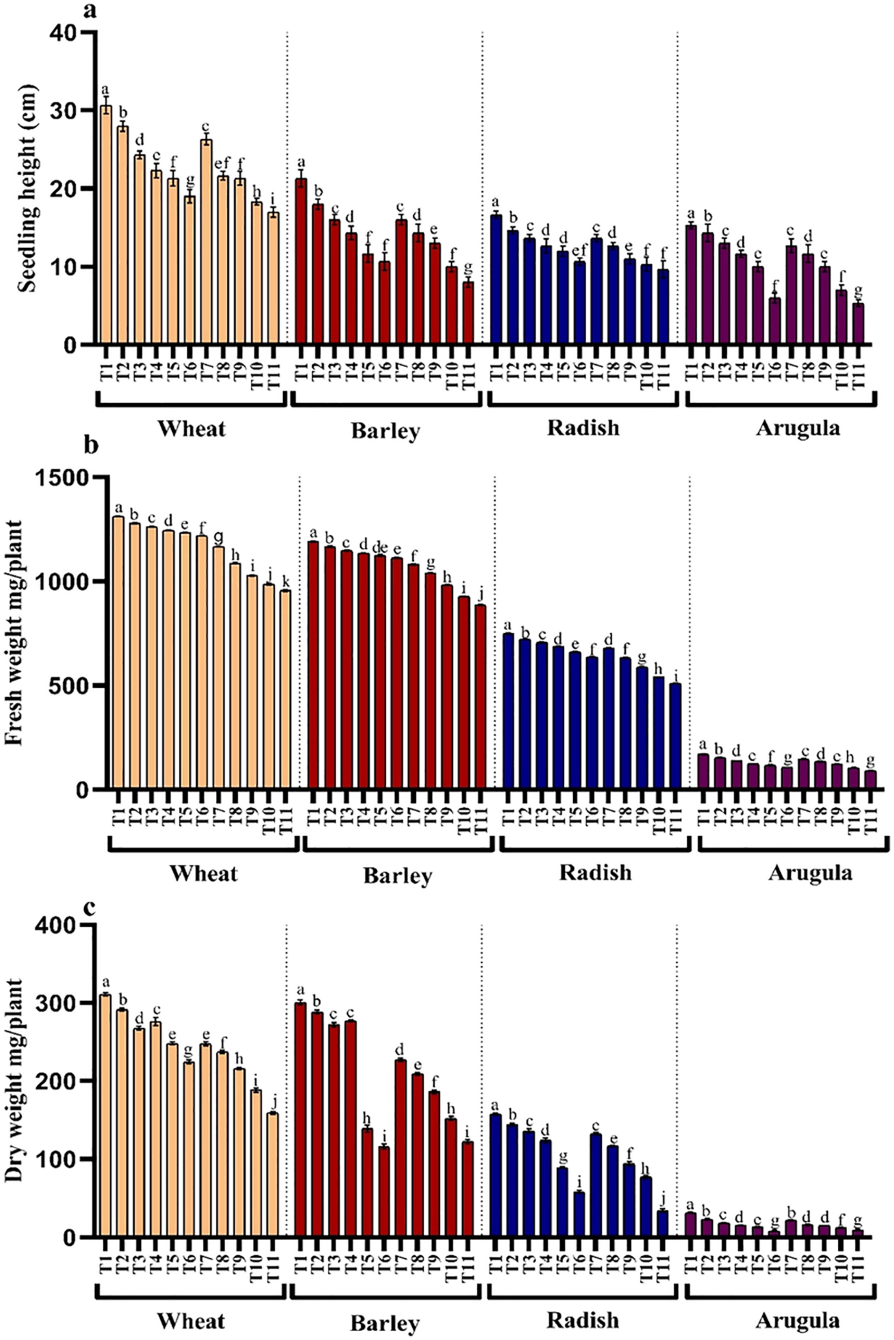
Effect of *A. Saligna* stem extracts (SE) and leaf extracts (LE) on (A) plant height, (B) fresh and (C) dry weight in wheat, barley, radish, and arugula plant. Data is mean (±SE) of three replicates, and different lowercase letters denote significant difference at *P* < 0.05. T1: control, T2: 5% SE, T3: 10% SE, T4: 15% SE, T5: 20% SE, T6: 25% SE, T7: 5% LE, T8: 10% LE, T9: 15% LE, T10: 20% LE, T11: 25% LE.

Seedling or plant height and fresh and dry weights were also significantly reduced with higher concentrations of the extracts in all the four tested plant species. However, leaf extracts (LE) proved more damaging than stem extracts (SE). Reduction showed a gradual trend with an increasing concentration of extracts. Relative to control, percent reduction in seedling height, fresh and dry weight was 44.55%, 27.13% and 48.87% for *T. aestivum*, 62.49%, 25.46% and 59.19% for *H.vulgare*, 42.01%, 31.84% and 78.23% for *R. sativus* and 65.23%, 46.06% and 70.53% for *E. Sativa* due to 25% LE treatment (Figs. 2A–2C).

### Photosynthetic pigments

**Figure 3:**
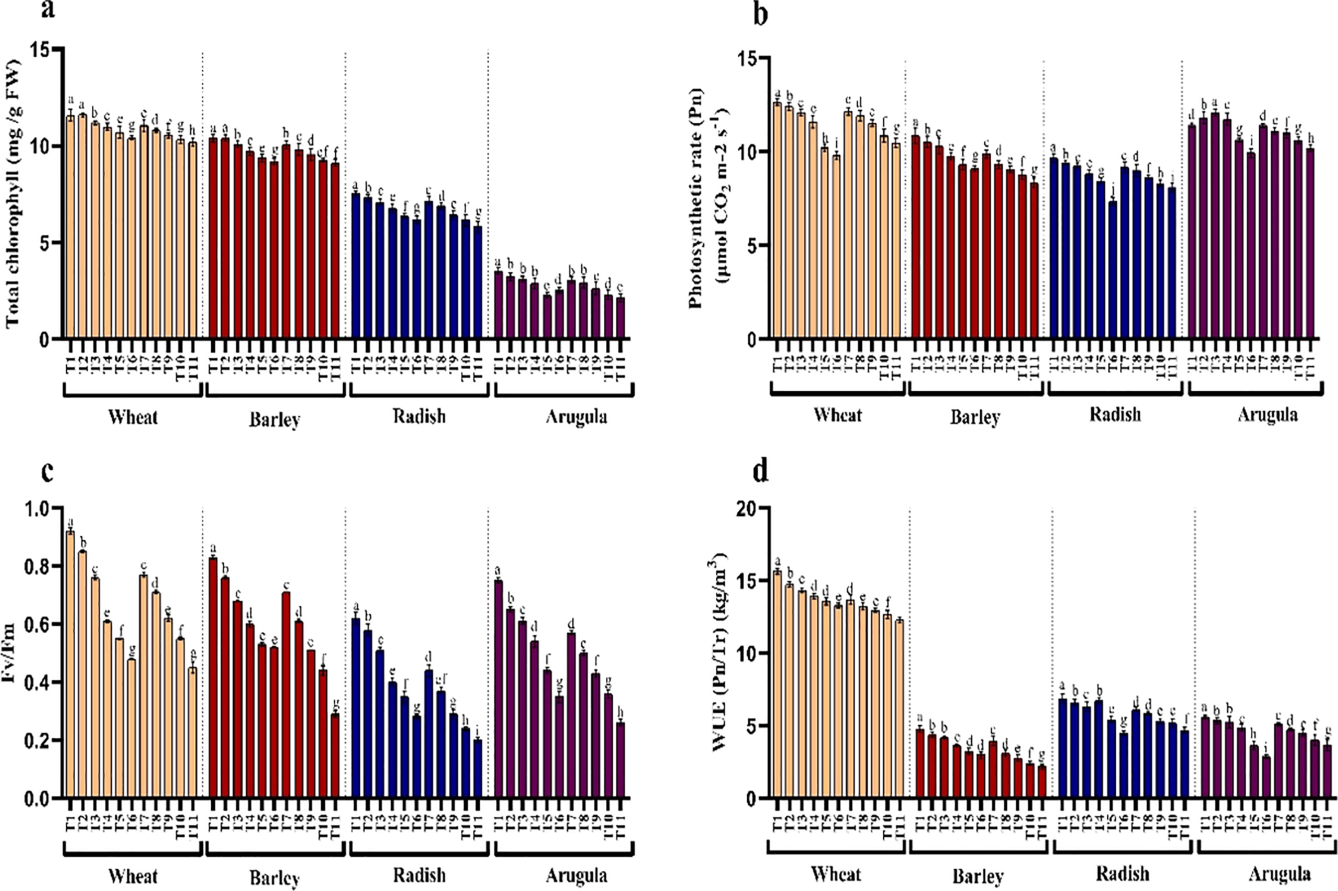
Effect of *A. Saligna* stem extracts (SE) and leaf extracts (LE) on (A) total chlorophyll, (B) net photosynthesis, (C) Fv/Fm, and (D) water use efficiency (WUE) in wheat, barley, radish, and arugula plant. Data is mean (±SE) of three replicates, and different lowercase letters denote significant difference at *P* < 0.05. T1: control, T2: 5% SE, T3: 10% SE, T4: 15% SE, T5: 20% SE, T6: 25% SE, T7: 5% LE, T8: 10% LE, T9: 15% LE, T10: 20% LE, T11: 25% LE.

The influence of SE and LE on the chlorophyll pigments, photosynthesis, and Fv/Fm activity in *T. aestivum, H*. vulgare, *R. sativus*, and *E. sativa* are shown in Figs. 3A–3C. Chlorophyll content decreased with increasing concentration of extracts attaining maximal reduction due to 25% concentration. At 25% of SE, LE of *A. saligna*, total chlorophylls were reduced by 10.10%, 12.09% in *T. aestivum*, 11.62%, 12.49% for *H. vulgare*, 17.80%, and 22.71% for *R. sativus* 28.24%, 39.09% for *E. Sativa*. Net photosynthesis and Fv/Fm activity in all four plant species was decreased maximally due to 25% SE and LE compared to control and other concentrations. Relative to control, the reduction in water use efficiency (WUE) was gradual with increasing concentrations of the extracts. The maximal decrease WUE was 15.16%, 21.37% for *T. aestivum*, 37.53%, 54.30% for *H.vulgare*, 34.79%, 31.88% for *R. sativus*, 48.57%, 34.64% for *E. Sativa* due to 25% of SE and LE respectively (Fig. 3D).

### H_2_O_2_, lipid peroxidation, and electrolyte leakage

**Figure 4:**
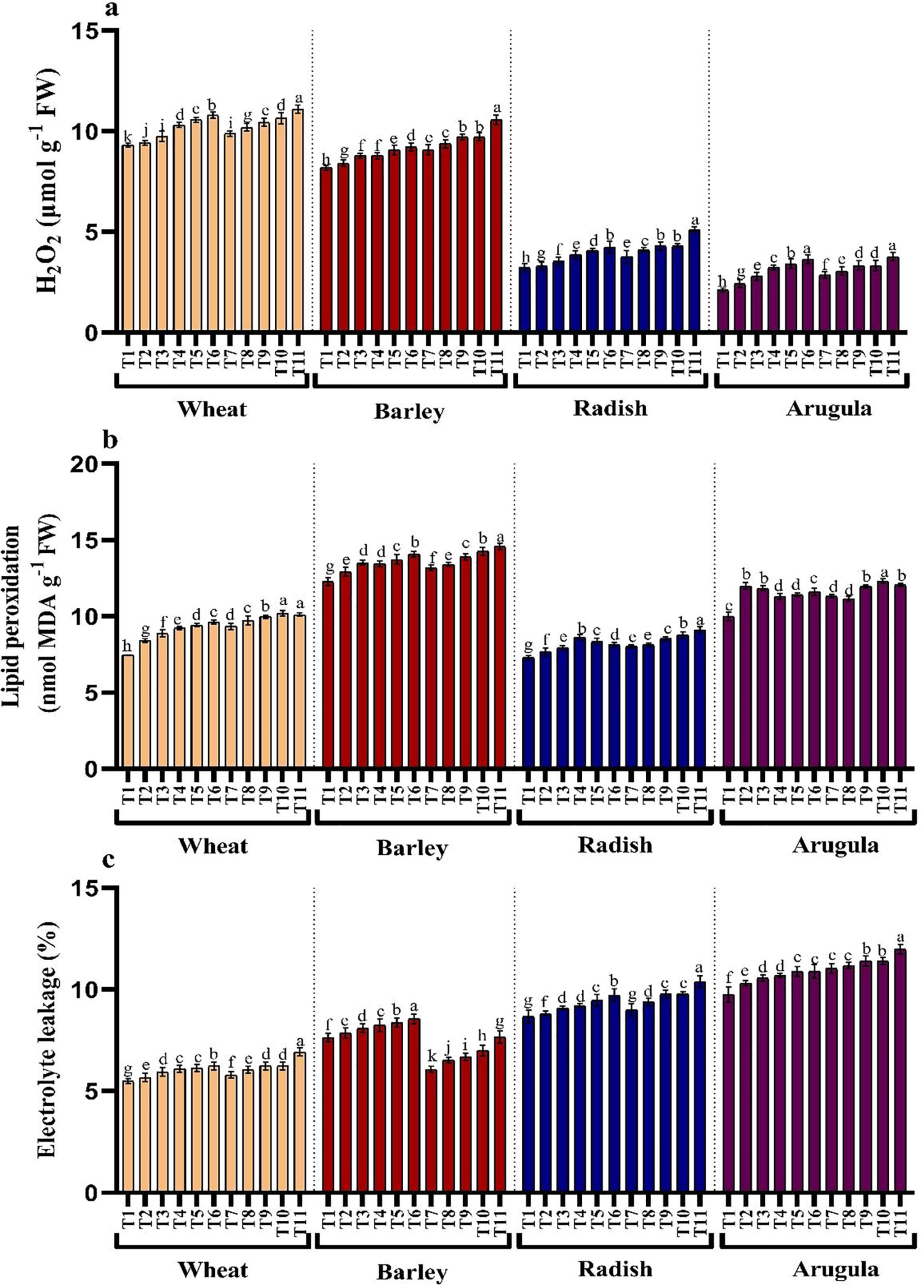
Effect of *A. Saligna* stem extracts (SE) and leaf extracts (LE) (A) hydrogen peroxide (H_2_O_2_), (B) lipid peroxidation, and (C) electrolyte leakage in wheat, barley, radish, and arugula plant. Data is mean (±SE) of three replicates, and different lowercase letters denote significant difference at *P* < 0.05. T1: control, T2: 5% SE, T3: 10% SE, T4: 15% SE, T5: 20% SE, T6: 25% SE, T7: 5% LE, T8: 10% LE, T9: 15% LE, T10: 20% LE, T11: 25% LE.

Treatments application from stem and leaf of *A. saligna* extracts on *T. aestivum, H*. vulgare, *R. sativus* and *E. sativa* resulted in induction of oxidative damage by triggering the generation of hydrogen peroxide (H_2_O_2_), resulting in lipid peroxidation and electrolyte leakage (Figs. 4A–4C). Relative to the control, H_2_O_2_ maximally increased by 16.18%, 19.46% for *T. aestivum*, 12.58%, 29.06% for *H.vulgare*, 31.27%, 58.51% for *R. sativus* 72.17%, 77.83% for *E. Sativa* due to 25% of SE and LE respectively (Fig. 3A). In addition, increased H_2_O_2_ accumulation resulted in increased lipid peroxidation exhibiting maximal increase with the treatment of 25% concentration. Electrolyte leakage also showed a noticeable increase in seedlings grown in the presence of extracts (Fig. 4C).

### Proline, soluble sugars, phenols, flavonoids, and protein

**Figure 5:**
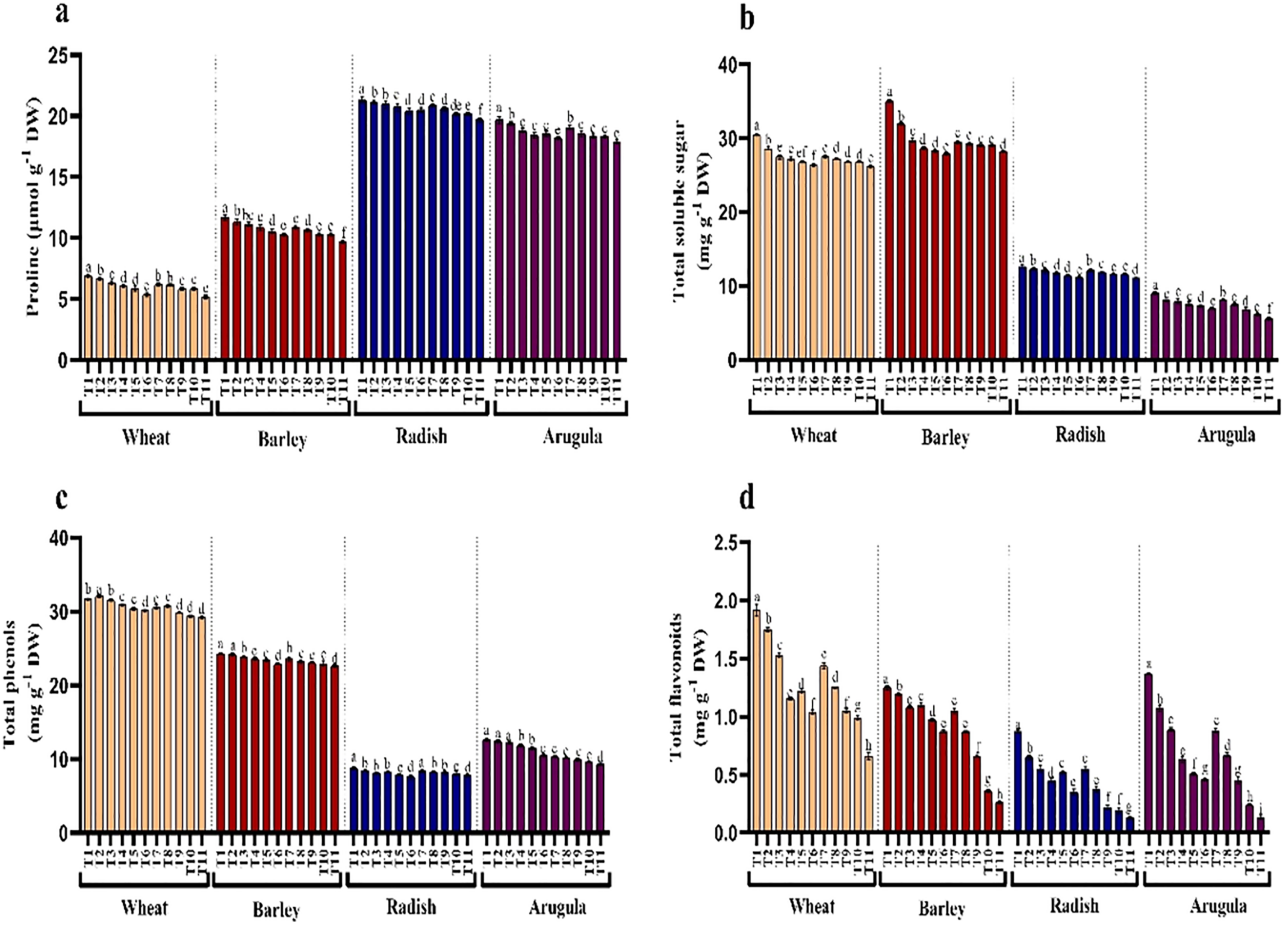
Effect of *A. Saligna* stem extracts (SE) and leaf extracts (LE) on (A) proline, (B) total soluble sugar, (C) total phenols, and (D) total flavonoids in wheat, barley, radish, and arugula plant. Data is mean (±SE) of three replicates, and different lowercase letters denote significant difference at *P* < 0.05. T1: control, T2: 5% SE, T3: 10% SE, T4: 15% SE, T5: 20% SE, T6: 25% SE, T7: 5% LE, T8: 10% LE, T9: 15% LE, T10: 20% LE, T11: 25% LE.

Seedlings treated with different extract concentrations of *A. saligna* exhibited decrease in the accumulation of proline and soluble sugars (Figs. 5A and 5B). Relative to control, treatment of both extracts induced gradual reduction with increasing concentrations imparting more obvious decrease at higher concentrations, *i.e*., 25%. It was observed that LE imparted more reduced than SE. In seedlings treated with 25% LE, the maximal decrease in proline was 25.18%, 17.05%, 7.70%, and 9.00%, and sugars were 13.92%, 13.64%, 12.35% and 32.69%, respectively in *T. aestivum, H*. vulgare, *R. sativus*, and *E. Sativa* (Figs. 5A and 5B).

Contents of total phenols and flavonoids decreased due to treatments of extracts in all four plant species. Relative to control, phenols and flavonoids decreased maximally in seedlings treated with 25% LE. Percent decrease observed was 7.74%, 65.68% in *T. aestivum*, 6.92%, 79.20% in *H.vulgare*, 11.29%, 85.06% in *R. sativus* 26.52%, 88.60% in *E. Sativa* for total phenols and total flavonoids respectively over the control (Figs. 5C and 5D).

### Protein content and antioxidant enzymes

**Figure 6:**
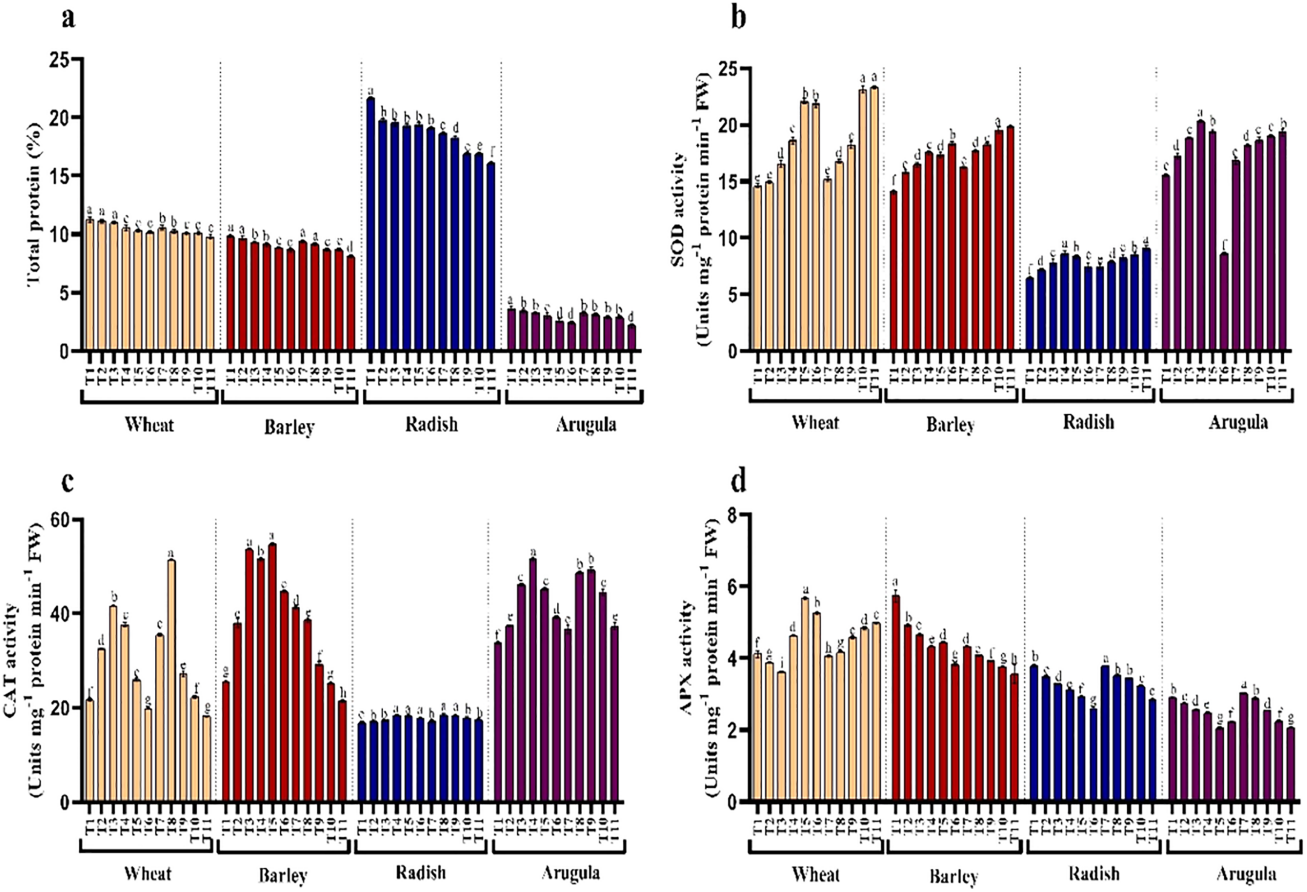
Effect of *A. Saligna* stem extracts (SE) and leaf extracts (LE) on (A) total protein content and activity of (B) SOD, (C) CAT, and (D) APX in wheat, barley, radish, and arugula plant. Data is mean (±SE) of three replicates, and different lowercase letters denote significant difference at *P* < 0.05. T1: control, T2: 5% SE, T3: 10% SE, T4: 15% SE, T5: 20% SE, T6: 25% SE, T7: 5% LE, T8: 10% LE, T9: 15% LE, T10: 20% LE, T11: 25% LE.

Seedlings treated with different extract concentrations of *A. saligna* exhibited a reduction in protein accumulation. For example, the maximal decrease in protein in seedlings treated with 25% LE, in *T. aestivum, H. vulgare, R. sativus, and E. Sativa* (Fig. 6A).

Seedlings of *T. aestivum, H. vulgare, R. sativus*, and *E. Sativa* that have grown with extracts of different concentrations showed differential responses for activity antioxidant enzymes assayed. Relative to control, SOD exhibited noticeable enhancement due to extracting treatments, while CAT and APX increased with lower concentrations while declining with higher concentrations of SE and LE.

### Relative gene expression

**Figure 7:**
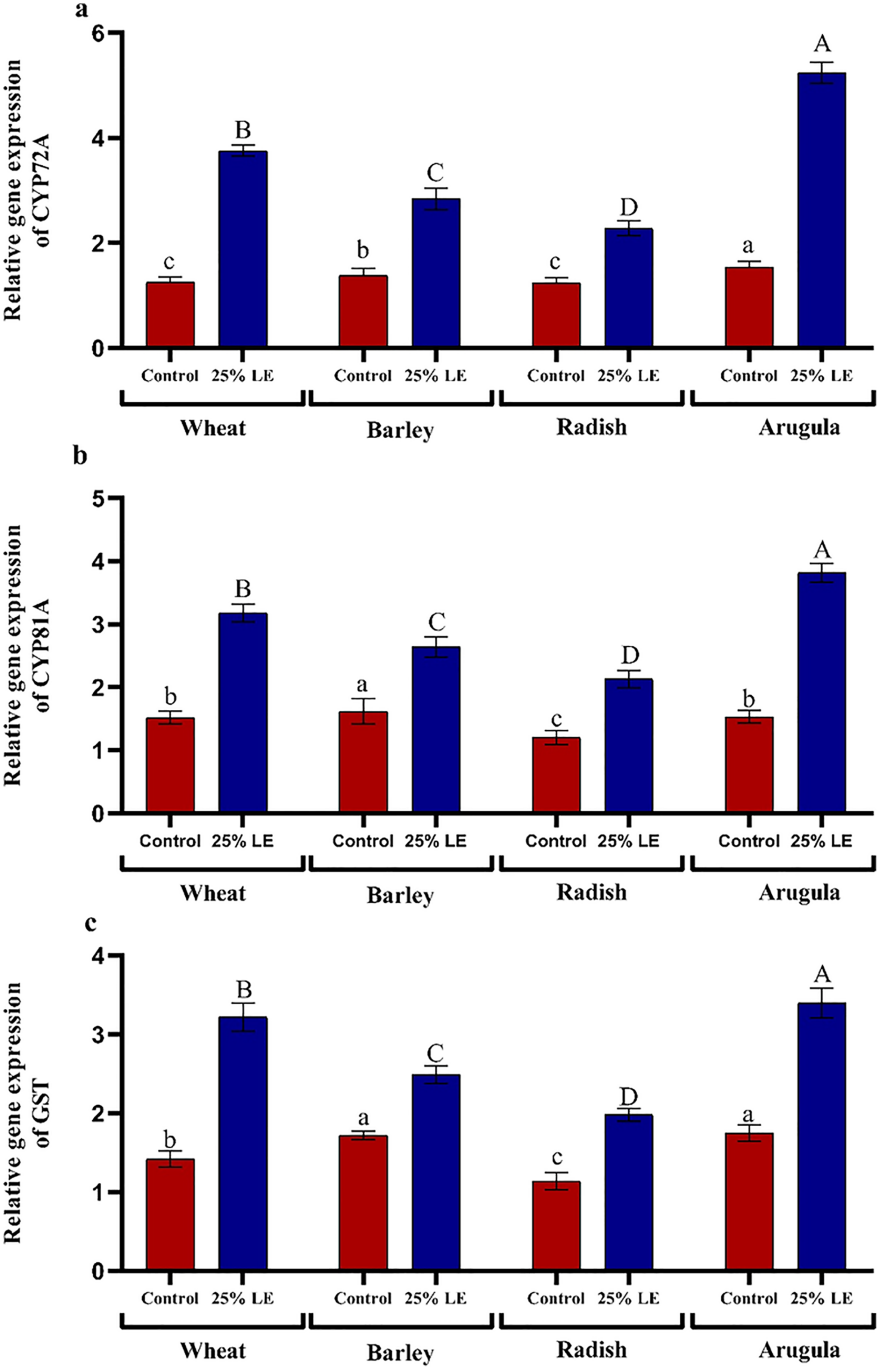
Effect of *A. Saligna* leaf extracts (25%) on the expression of (A) CYP72A, (B) CYP81A, and (C) GST in wheat, barley, radish, and arugula plant. Data is mean (±SE) of three replicates, and different lowercase letters denote significant difference at *P* < 0.05.

Treatment of *A. saligna* extracts on *T. aestivum, H. vulgare, R. sativus*, and *E. sativa* seedlings had increased gene expression of CYP72A, CYP81A, and GST. The relative gene expression of CYP72A was greater in extract treated plants as compared to control plants. In case of CYP81A expression, the 25% LE increased gene expression in *T. aestivum, H. vulgare, R. sativus*, and *E. sativa* seedlings as compared to their respective controls. A similar results were observed in GST gene expression in which maximum gene expression was observed in *E. sativa* followed by *T. aestivum*, *R. sativus*, and *H. vulgare*, with respect to their respective control treatments (Figs. 7A–7C).

## Discussion

Phytoxicity imparted by the neighboring plant species is one of the main causes for the maximum reduction in growth and yield of crops. The current study investigated the influence of extracts from *A. saligna* on the growth of *T. aestivum, H. vulgare, R. sativus*, and *E. Sativa*. It was observed that extracts induced a significant decrease in germination and the other studied parameters of tested crops. Extracts from stem and leaf showed differential effects on the tested crops, with leaf extracts imparting many damaging effects. It has been reported that plant parts differ in the content of allelochemicals (Argal et al., 2016). Therefore, it can be concluded that leaf extracts may have higher concentrations of allelochemicals or have the presence of some exclusive allelochemicals. According to Fernandez et al. (2009), the allelochemicals are presented higher in leaves than roots. Allelochemicals mostly affect root growth because of their direct contact. Our study observed that higher concentrations of extracts influenced the growth in terms of length, weight, and biomass accumulation for tested crops seedlings. Inderjit (1996) has attributed the inhibitory effect of allelochemicals to their high-water solubility. It has been accepted that phenols form the key allelochemicals inhibiting the growth of plants (Blum et al., 1993). Reduction in germination and growth of mustard, millet, tomato, mung bean, corn, and radish is due to the release of phenolic compounds like vanillic acid, catechol, gallic acid, and syringic acid from *Cornus canadensis* L. (Shaukat, Munir & Siddiqui, 2003). Plant extracts can prove inhibitory or stimulatory effect based on their concentration, and the current study also showed that extracts of *A. saligna* imparted slight inhibitory effects even at a lower concentration. However, its impact was much evident at higher concentrations. In addition, *E. Sativa* exhibited more sensitivity to *A. saligna* extracts as compared to other tested crops. Growth inhibition of plants by allelochemicals can be considered a synergistic impact (Fagg & Stewart, 1994).

Treatment with the extracts of *A. saligna* reduced the chlorophylls contents, photosynthetic rate, WUE, and PSII activity (Fv/Fm), and maximal reduction in seedlings was recorded that were treated with higher concentrations of shoot and leaf extracts. It was in great analogy with our results that reduction in green contents was caused by plant extract treatment (Abou El-Ghit, 2016; Singh, Pandey & Singh, 2009; Singh, Singh & Singh, 2009; Tomar, Sharma & Agarwal, 2015). Reduced Pn and Fv/Fm in extract-treated seedlings reflect the stomatal and non-stomatal photosynthetic restriction. Earlier Singh, Pandey & Singh (2009) also reported reduced photosynthesis in bananas after application of *Cyperus rotundus* extracts. Reduced photosynthesis and PSII activity in tested crops due to *A. saligna* leachate treatment was correlated with reduced WUE, which can significantly impact the yield productivity.

Seedlings of all four plants treated with extract of *A. saligna* exhibited increased generation and accumulation of free radicals like H_2_O_2_. Leachate treatments enhance the generation of toxic free radicals (El-Sheshtawy et al., 2021; Howladar, 2014). Excessive generation of ROS like singlet oxygen, H_2_O_2_, hydroxyl radical leads to the oxidative damage to the key macromolecules like proteins, lipids, and nucleic acids (Ahanger et al., 2018; Abdel Maksoud et al., 2022). Such damage led by excess ROS may reduce the membrane stability, thereby leading to the electrolyte leakage, which was obvious in this investigation. In a few other studies, increased generation of ROS due to leachate treatment (Cruz-Ortega, Ayala-Cordero & Anaya, 2002; Oracz et al., 2007). Stress-induced lipid peroxidation and electrolyte leakage are considered key parameters for assessing crop plants’ stress intensity and tolerance levels (Ahanger et al., 2020a; Ahanger et al., 2020b). Seedlings treated with higher concentrations of *A. saligna* extracts resulted in increased electrolyte leakage and lipid peroxidation due to loss of membrane integrity and functioning. Loss of structural and functional integrity of membranes due to treatment of plant extracts has been reported by Alqarawi et al. (2018). Allelopathic extracts reduced the concentration of polyunsaturated fatty acids resulting in loss of membrane structure and functioning (Alqarawi et al., 2018). It has been reported that stressful growth conditions trigger the activity of lipoxygenase and the generation of ROS, resulting in increased lipid peroxidation and membrane damage (Ahanger et al., 2019; Megahed et al., 2013). Increased ROS accumulation reduced the photosynthetic functioning by affecting the structure and function of chloroplast machinery (Bi et al., 2009; Shapiguzov et al., 2012). Plants *Lycopersicum esculentum* treated with aqueous extract of *Sicyos deppei* resulted in enhancement in production of ROS, such as hydrogen peroxide and superoxide concomitant due to increased activity of NADPH-oxidase (Zhang et al., 2010). P-coumaric and vanillic acid have induced the generation of O_2_- radicals in *Microcystis aeruginosa* cells (Bi et al., 2009). According to Agha et al. (2021), crop plants exposed to environmental stresses result in more damage due to ROS production. Stresses increase the activity of radical generating plasma membrane oxidase, NADP-oxidase (El-Sheshtawy et al., 2022). Advance research is required to investigate the actual process leading to increased ROS generation under allelochemical stress.

For averting the damage caused due to excessive generation of ROS, plants up-regulate the tolerance mechanisms aimed at scavenging the excess ROS. In the present study, *A. saligna* extract treatment led to the differential up-regulation of the antioxidant enzyme activities. The activity of antioxidant enzymes including SOD, CAT, and APX assayed showed different responses, with SOD exhibiting apparent increase with increasing concentration of extracts while CAT exhibited increase with a lower concentration and slight decrease with higher concentration. SOD scavenges superoxide radicals, thereby protecting photosynthetic electron transport, while hydrogen peroxide is neutralized by CAT in the cytosol or by APX in chloroplast and mitochondria *via* ascorbate-glutathione cycle (Ahanger et al., 2017; El-Sheshtawy et al., 2022). Environmental stresses, including biotic and abiotic components like drought, salinity, extreme temperatures, metals, metalloids, allelopathic stress, and disease infestation, result in increased ROS generation and altered cellular functioning (Ahanger et al., 2020a; Ahanger et al., 2017; Alqarawi et al., 2018; Khan et al., 2015; Parvaiz, 2012). Comparable to our findings, Singh, Singh & Singh (2009) have also revealed differential regulation of antioxidant system in *Zea mays* due to application of aqueous extracts of *Nicotiana plumbaginifolia*. Greater stress tolerance in plants is associated with more antioxidant functioning. Antioxidant enzymes like SOD, CAT, and APX work closely with non-enzymatic components to neutralize the toxic radicals produced under stress (Shapiguzov et al., 2012). Cruz-Ortega, Ayala-Cordero & Anaya (2002) have reported increased CAT activity in bean, maize, and tomato treated with *Callicarpa acuminate* extracts. It has been reported that the presence of water-soluble phenolics in leaf extract of sunflower imparted oxidative stress in mustard by triggering excess ROS generation resulting in increased activity of SOD and CAT (Oracz et al., 2007). Batish et al. (2007) have demonstrated increased ROS generation in mung beans due to caffeic acid treatment resulting in a significant alteration in the activity of peroxidases. Increased activity of SOD and POD protects membranes and cellular functioning by preventing the formation of toxic hydroxyl radicals. The presence of a higher concentration of allelochemical-like phenolic compounds like p-hydroxybenzoic acid and ferulic acid can induce oxidative stress (Zhang et al., 2010) by increasing the generation of free radicals’ initiation of antioxidants (Abu-Shahba et al., 2022; Lara-NuÑez et al., 2006). Under intense oxidative stress, excessive ROS generation may cause a decrease in antioxidant enzymes’ activity, which may be attributed to the presence of higher contents of allelochemicals. ROS mediate signal transduction when present in optimal concentration; however, it can prove deleterious for metabolism when the concentration exceeds the antioxidants’ scavenging potential, leading to programmed cell death (Sofy et al., 2022).

Seedlings treated with aqueous extracts of *A. saligna* exhibited a decrease in compatible osmolytes consisting of free sugar and proline, with the effect being much obvious at higher (25%) concentrations. Similar to our results earlier, Singh, Singh & Singh (2009) has demonstrated reduced sugar accumulation in maize due to applying aqueous extracts of *Nicotiana plumbaginifolia*. Similarly, Rao & Singh (2015) reported a significant decrease in the synthesis of sugars in pea seedlings treated with *Hyptis suaveolens* L leachates. In maize and *Phaseolus vulgaris*, treatment of higher concentration of leaf leachates of *Acacia nilotica* and *Eucalyptus rostrata* has been reported to induce the expression of stress proteins and ABA formation (El-Khawas & Shehata, 2005). Accumulation of osmolytes, including amino acid, sugar, and proline, contribute to turgor maintenance under stressful conditions (Ahanger et al., 2014; Fouda & Sofy, 2022). Accumulation of compatible solutes assists in counteracting the unfavorable conditions by protecting membrane structural and functional integrity, enzyme functioning through osmotic adjustment (Megahed et al., 2012; Sivakumar et al., 2002). However, the reduced accumulation of proline and sugars observed in the present study reflects the damaging effects of *A. saligna* extracts on the tested crop species. In addition to their role as an osmolyte, proline and sugars are believed to assist in ROS scavenging (Ahanger et al., 2018; Hayat et al., 2012).

The CYP gene family is among the large families involved in the development of plants and mediates the synthesis of key secondary signals (Biazzi et al., 2015). In the present study, 25% leaf extract treated seedlings significantly enhanced CYP72A, CYP81A, and GST gene expression. GST genes are ubiquitous and have an important role in stress mitigation (Gullner et al., 2018). However, studies discussing the influence of plant extracts on the expression of CYP and GST are not available. Increased expression of GST prevents cytotoxic effects of stresses, mediates detoxification of xenobiotics, thereby restricting the damage to macromolecules (Sytykiewicz, 2011).

## Conclusion

In conclusion, the extracts of *A. saligna* deleteriously affected the growth of wheat, barley, radish, and arugula by reducing germination, and promoting the accumulation of osmolytes, and secondary metabolism. Allelopathic effects were further obvious as reduced membrane functioning reflected as increased lipid peroxidation due to ROS production. Differential regulation of antioxidant enzymes and increased gene expression of CYP and GST were also evident. The present studies indicated that each tested crop exhibited reduced germination rate, plant height, fresh and dry weight with the application of *A. saligna* extracts. Moreover, the activities of antioxidant enzymes, including superoxide dismutase (SOD), catalase (CAT), and ascorbate peroxidase (APX), exhibited varying regulation due to the extracts application. Higher concentration of A. Saligna dry leachates reduced chlorophyll content, photosynthesis, PSII activity, and water use efficiency. Furthermore, the content of proline, sugars, protein, total phenols, and flavonoids were also decreased considerably due to the instant extracts application. On other hand high concentrations of LE enhanced the expression of genes. The study concludes the presence of significant allelochemicals in *Acaccia saligna*. Therefore, further studies are suggested to explore the natural compounds in *A. saligna* imparting such inhibitory effects on the plants.

## Supplemental Information

10.7717/peerj.13623/supp-1 Supplemental Information 1 Raw data.

## Additional Information and Declarations

### Data Availability

The following information was supplied regarding data availability: The raw data is available in the Supplemental File.
